# Effects of Biogents Sentinel Trap Field Placement on Capture Rates of Adult Asian Tiger Mosquitoes, *Aedes albopictus*

**DOI:** 10.1371/journal.pone.0060524

**Published:** 2013-03-29

**Authors:** Taryn N. Crepeau, Sean P. Healy, Kristen Bartlett-Healy, Isik Unlu, Ary Farajollahi, Dina M. Fonseca

**Affiliations:** 1 Monmouth County Mosquito Extermination Commission, Eatontown, New Jersey, United States of America; 2 Center for Vector Biology, Rutgers University, New Brunswick, New Jersey, United States of America; 3 Mercer County Mosquito Control, West Trenton, New Jersey, United States of America; Institut Pasteur, France

## Abstract

The Biogents® Sentinel (BGS) trap is the standard tool to monitor adult *Aedes* (*Stegomyia*) *albopictus* (Skuse) (Diptera: Culicidae), the Asian tiger mosquito. BGS traps are commonly placed in residential properties during surveillance operations, but locations within properties may have significant differences in ambient light, temperature, and humidity (e.g. between a sunlit lawn and shady underbrush). We examined the effect of BGS trap placement on *Ae. albopictus* capture rates in three residential properties in Monmouth County, New Jersey, USA. In each property we visually selected locations as shade, partial shade, and sun. Traps in “partial shade” locations were under vegetation and were exposed to filtered sunlight during some parts of the day while “shaded” locations were never exposed to direct sunlight. Locations defined as “sun” were exposed to direct sunlight for large parts of the day. We placed a BGS trap in each of the three location types and used small data loggers to measure temperature, relative humidity, and light exposure at each trap during a 24-hour deployment. To address temporal variability, we made seven separate measurements from 31 August to 22 September 2010. We found that “partial shade” and “full shade” locations did not differ but that “full sun” locations had significantly higher light exposure, higher temperature, and lower humidity. Importantly, *Ae. albopictus* catches (males, females, or both) were consistently and significantly over 3 times higher in traps located in shaded locations. To further investigate the effects of local temperature and humidity on surveillance we examined *Ae. albopictus* collections from 37 BGS traps fitted with data loggers and deployed weekly from August through mid October, during the 2009 season, in three urban sites in Mercer County, NJ. We confirmed that local climate influences capture rates and that *Ae. albopictus* surveillance projects need to monitor trap placement carefully for maximum efficiency.

## Introduction

Good surveillance is the foundation of informative field epidemiology and effective vector or nuisance mosquito control programs. The BG Sentinel™ trap (BGS trap, Biogents AG, Regensburg, Germany) has become the established tool to survey *Aedes* (*Stegomyia*) *albopictus* (Skuse 1895), the Asian tiger mosquito (ATM), a daytime biting species. The effectiveness of this trap has been demonstrated for temperate populations of *Ae. albopictus* [1], [2] but the effect of specific environmental characteristics in the location of the traps has not been evaluated. Adult mosquito surveillance has historically focused on night and crepuscular biting mosquitoes [3]. While at those times temperature and humidity are relatively evenly distributed across the sites being surveyed, direct sunlight during the day creates a patchwork of temperatures and humidity. In addition, adult *Ae. albopictus* in general are thought to be highly sensitive to low humidity conditions [4], [5] and therefore the interplay between ambient light, temperature, and humidity are of notable importance.

*Aedes albopictus* is native to Asia but has spread to at least 28 countries outside its native range and to all continents except Antarctica [6], [7]. In its exotic range, this container-inhabiting mosquito is strongly associated with humans and occurs primarily in urban and suburban areas where it exploits small artificial containers such as buckets, plant saucers, and tires [4], [5], [8]. Since this species readily uses tires as oviposition sites, the used tire trade has become a primary means for the global spread of *Ae. albopictus* [9], although the trade of small potted plants with water reservoirs, such as “lucky bamboo” (*Dracaena* spp.) has also been implicated [10], [11]. *Aedes albopictus* was responsible for the 2001–2002 epidemic of dengue fever in Hawaii [12], although elsewhere in the USA this species is mostly considered just an important nuisance [13]. Recently, however, a single base pair mutation in the chikungunya virus (CHIKV) increased its susceptibility to transmission by *Ae. albopictus* [14], [15] and this mosquito has become the principal vector in a large (>1,000,0000 cases) epidemic of chikungunya fever in the Indian Ocean Basin and Africa that started in 2004 [16]. Although chikungunya fever has not spread broadly in the temperate zone, an epidemic in northern Italy in 2007 sickened over 200 people [17] and small numbers of locally transmitted CHIKV cases have been identified in southern France since 2009 [18].

Our study is part of an area-wide project for the management of the Asian tiger mosquito (AW-ATM), which aims to examine the area-wide efficacy and sustainability of existing strategies for reducing the nuisance and public health threat posed by *Ae. albopictus*. The project is based on detailed comparisons between previously defined groups of 1,000 parcels ( = single-home and surrounding yard) in urban and suburban settings in two counties in the state of New Jersey, USA [19]. The objective of this study was to examine the impact of trap location on the capture rate of *Ae. albopictus* with BGS traps and how variation in temperature and humidity at fine spatial scales may affect surveillance of this day-biting species.

## Materials and Methods

### Ethics statement

No specific permits were required for the described field studies, which were developed with homeowner consent by professional county mosquito control personnel. These studies did not involve endangered or protected species.

### Experimental test of the effect of trap placement on *Aedes albopictus* catches

We chose three suburban residential locations in Union Beach Borough, Monmouth County, NJ, for this study that took place from 31 August through 28 October 2010. During this time a total of 14 trapping events took place. We visually identified areas at these residences that qualified as one of three experimental treatments [20]: shade, partial shade, and sun. We defined “shaded” locations as those where the trap was never exposed to direct sunlight; “partial shade” locations were under vegetation and were exposed to filtered sunlight during some parts of the day; locations defined as “sun” were exposed to direct sunlight for large parts of the day. One BGS trap was placed in each of the three treatments within each of the three residences, for a total of nine traps per trapping event. BGS traps were baited with a standard Biogents® BG-Lure (Biogents AG, Regensburg, Germany) and were deployed for approximately 24 hours. Deployment time depended on travel time and personnel availability but all traps (all treatments) in each residence were collected within minutes of each other, and all nine traps were retrieved within 1 hour. Trap catches were transported to the laboratory, identified to species, and counted. Numbers of female and male *Ae. albopictus* were recorded separately.

To test for differences in light, temperature, and humidity between treatments, each trap was fitted with a HOBO® Pendant Temperature/Light Data Logger (Onset Computer Corporation, Pocasset, MA, USA) that measures light and temperature at set time intervals and an iButton® Hygrochron™ Temperature/Humidity Logger (Maxim Integrated Products, Inc. Sunnyvale, CA, USA) that measures relative humidity and temperature. Both devices were set to take measurements every 5 minutes. The HOBO data logger is approximately 2.5 cm wide ×5 cm long ×1.5 cm high and is enclosed in a clear plastic waterproof case. The Hygrochron is a small round stainless steel canister, approximately 17 mm in diameter and 7 mm in height, which encases a microchip. To eliminate equipment bias, ten sets of equipment (BGS trap with lure, HOBO data logger, and Hygrochron) were available and each week nine were assigned randomly to residences and light treatments.

**Figure 1:**
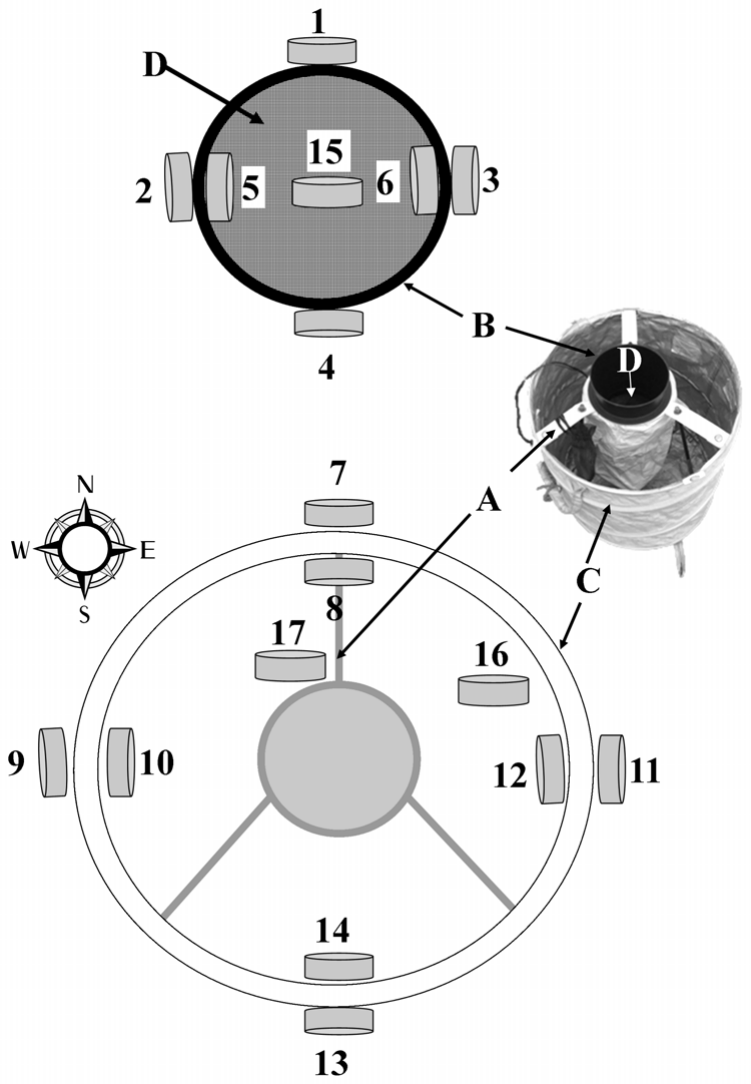
Locations of Hygrochrons within and on the Biogents BG-Sentinel trap. A = support bar, B = black catch pipe, C = white plastic trap, and D = catch net affixed to the bottom of the black catch pipe. Locations 1–6 were affixed to the catch pipe with adhesive, Locations 7–14 and 17 were placed inside small hanging mesh pouches attached from the support bar. Location 15 was placed inside the catch net. Location 16 was placed on the bottom of the white plastic trap 1” from the side. Traps were always positioned with location 17 facing north (support bar labeled A) as in the schematic diagram on the bottom of the figure. Please note that the small inset picture of a BGS trap is positioned for ease of labeling and is not facing correctly with respect to the cardinal rose.

We placed the HOBO data loggers on top of the trap with the “bottom” of the logger facing east for consistency of measurement. This placement allowed incident light to reach the unit directly, a necessity for light measurement. However, this led to large spikes in temperature as the unit was heated by the direct exposure to sunlight, making this device at this location an inaccurate means of temperature measurement. In contrast, the Hygrochron were placed in a small mesh pouch hanging inside the trap protected from direct incident light. This location was chosen after we examined the effect of the placement of the Hygrochrons at 17 different locations in or on a BGS trap. To do this we fitted 3 separate BGS traps with 17 Hygrochrons each (please refer to Figure 1 for details of each location), placed the 3 traps in a triangular array (a equilateral triangle 10 m on each side) in an open field, and set each Hygrochron to measure temperature and relative humidity every 30 min for 24 hours. This test was performed once and each BGS trap represents a replicate. All three traps were oriented identically so the crossbar with the bag holding a Hygrochron at position#17 was always pointing north (please refer to Figure 1 for more details).

### Examination of the effect of local temperature and humidity on *Aedes albopictus* surveillance

During the 2009 *Ae. albopictus* active season (from May to October) we operated 37 BGS traps in 3 groups of 1,000 residences in the City of Trenton, Mercer County, NJ [21], 15 in an untreated site, 10 in a site where only source reduction through education was implemented, and 12 in a site where several integrated mosquito management strategies were implemented. Further details on trapping methods and control protocols during the 2009 AW-ATM field season can be found in Fonseca and colleagues [21]. We instructed AW-ATM field staff to deploy traps in areas sheltered by vegetation or manmade structures and placed Hygrochrons in position#17 inside a small mesh bag in all BGS traps during deployment. Traps were deployed weekly for approximately 24 hours but since surveillance teams placed each trap in succession they remained in the field for specific time periods that differed by 10 minutes to a few hours. Therefore, the Hygrochrons were set to measure temperature and relative humidity at 1-minute intervals to facilitate comparisons of identical time periods. At the end of each trapping event, data from each Hygrochron were downloaded into spreadsheets. To make meaningful comparisons across traps, we selected temperature and humidity readings (the response variable) during a subset of time intervals when all traps were running. Specifically, we determined that all traps were operating between 15:00 and 15:59 on day 1 and between 07:00 and 07:59 on day 2, and restricted the analyses to those two 60-minute periods. Over 1.5 million temperature and relative humidity readings were recorded for the season but when a BGS trap failed the environmental data associated with that trap were eliminated from the analysis. Three BGS traps failed on average per trapping date in Mercer (mean±SD = 3.1±1.5), therefore the sample size ranged from 31–36 with losses evenly distributed among the 3 sites. Please refer to other publications from our group [22], [23] for a detailed assessment of rate of BGS trap failure and practical ways to eliminate this problem.

### Statistical Analyses

The effect of the placement of the Hygrochron in and around each BGS trap on temperature and humidity measurements was analyzed with a repeated measures multivariate analysis of variance (JMP 8™, SAS Institute, Cary, NC) nested within each trap. Upon examination, all nighttime values were very similar therefore we constrained the analysis to 20 time points during 10 daytime hours (12:00–18:00 and 08:00–12:00) to reduce the overall variance. The use of data from only daylight hours is further justified by the fact that *Ae. albopictus* is a day-biting species. We used repeated measures multivariate analysis of variance to examine differences among the three treatments (Sun, Partial Shade, and Shade) in incident light, temperature, and capture rates of male and female adult *Ae. albopictus*. Because missing data significantly undermine a repeated measures analyses [24], failures in the HOBO, Hygrochron, or BGS traps forced us to remove 7 of the 14 trapping days and analyze only a total of 7 measurements between 31 August 31 and 22 September 2009 for each of the 9 traps (3 locations per residence ×3 replicate residences). Because no significant differences in light were found between Shade and Partial Shade these treatments were combined in the analysis of relative humidity.

For the large-scale trap deployment we restricted our analysis of the effects of temperature and humidity on catch rates to the 15:00 hr measurements (see results for details). The correlations between local temperature and humidity measured at 15:00 hr and log_10_ transformed *Ae. albopictus* captures were analyzed separately for each trapping day using a standard least squares regression nested within Date. To remove the effects of large numbers of zeros, we also restricted the analyses to 16 weekly collections from the end of July to mid-October (29 July to 15 October 2009), during the peak *Ae. albopictus* season that year.

## Results

**Figure 2:**
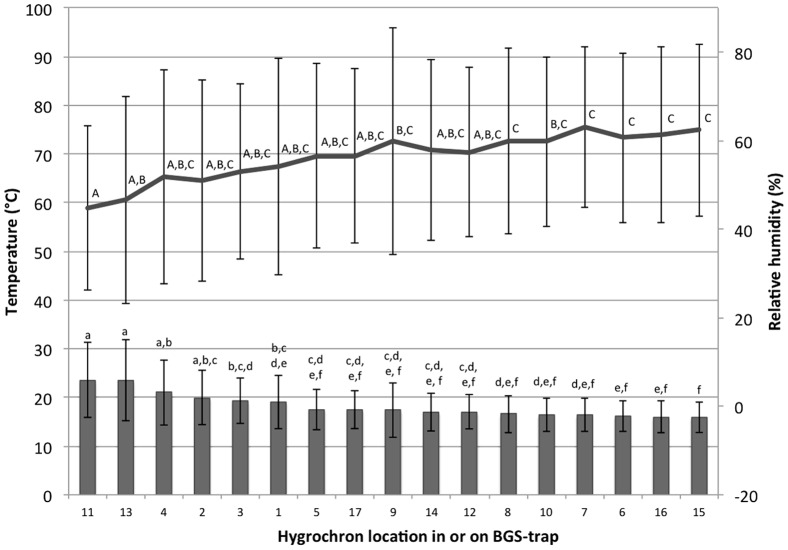
Mean temperature (bars) and mean relative humidity (line) of Hygrochron locations. Error bars are ±1 standard deviation. Levels not connected by the same letter are significantly different (based on a Least Squares Means Differences Tukey-Kramer Honestly Significant Difference test which accounts for multiple comparisons).

Hygrochrons placed in different locations in and on the three experimental BGS traps registered significantly different values of temperature (F_16_,_33_ ratio  = 66.0, P<0.0001) and relative humidity (RH, F_16_,_33_ ratio  = 27.81, P<0.0001), but repeated measures comparisons among the three replicates did not reveal differences (F_2,47_ = 0.05, P = 0.95; F_2,47_ = 0.63, P = 0.54, for temperature and RH, respectively). The Hygrochrons that were placed on the outside of the traps or affixed on the outside of the black catch pipe facing south and east and therefore exposed to direct light for longer periods of time (positions #11, 13, Figure 1) registered the most extreme temperatures and lowest RH (Figure 2). Hygrochrons attached to the catch pipe with tape (position# 1, 2, 3, 4) registered relatively high temperatures possibly the result of heat radiating from the black surface. In contrast, those inside the catch pipe (especially #15 that was in a pouch) registered consistently low temperatures possibly due to the homogenizing effects of airflow created by the fan (located only a few centimeters below). Position# 17, where the Hygrochron was placed inside a mesh bag hanging from the central support bar generated values in the middle range and with low variance (Figure 2). This location and method of attachment also allowed for the best security of the device and ease of deployment and therefore was selected as the standard location for placement of Hygrochrons in all experiments and surveillance.

We found significant differences in incident light between treatments (Sun = 29,920±2,451 lux; Partial Shade = 6,719±1,136 lux; shade = 4,393±1,637 lux, F_2,6_ = 18.05, P<0.01), although Partial Shade and Shade did not differ significantly from each other. Similarly, Partial Shade and Shade treatments had significantly higher relative humidity than Sun treatments (Sun = 59.5±1.6%, Partial shade = 62±2.2%, Shade = 61.6±2.2%, F_1,5,_ = 11.6, P = 0.01) but did not differ from each other. As mentioned, to increase statistical power [25] we combined the two shaded treatments into one and nested the comparisons of RH within residences since there were differences between replicates in average RH (F_2,5_ = 13.19, P = 0.01). There were also significant differences in temperature between all three treatments measured with the Hygrochron (Sun = 23.6±−0.9°C, Partial Shade = 22.6±0.86°C, Shade = 22.5±0.87°C, F_2,6_ = 7.51, P = 0.02), and the HOBO data logger (Sun = 25.1±0.89°C, Partial shade = 23.4±1.03°C, Shade = 22.5±0.89°C, F_2,6_ = 11.33, P<0.01). As expected, the differences are larger when temperatures were measured with the HOBO data logger due to the effect of direct exposure to sunlight. During each trapping event, incident light (measured with the HOBO) and temperature (measured with a Hygrochron at position #17) were highly and significantly correlated (r = 0.99, P<0.001, nested ANOVA, F_7,13_ = 10.91, P<0.0001).

**Figure 3:**
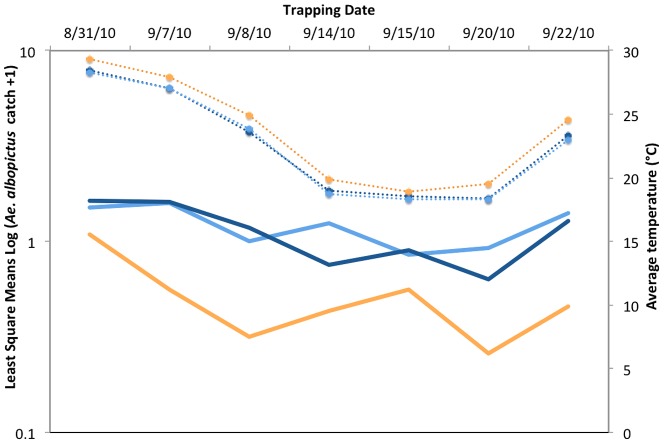
Comparison of trap catches in the three treatments over time. Orange = Sun, medium blue = Partial Shade, dark blue = Shade. Superimposed are average temperatures for each treatment (dashed lines, same color code).

We found that total (male + female) adult *Ae. albopictus* catches in “full shade” and “partial shade” treatments were significantly higher than in “full sun”, but not different from each other (Sun = 4.14±1.2, Partial shade = 21.7±3.7, Shade = 21.4±4.8, F_2,6_ = 11.22, P<0.01, Figure 3). This was also true for female and male catches analyzed separately (data not shown for brevity). Of note, although above we report average mosquito numbers, prior to analyses trap captures were log_10_(x+1) transformed to achieve normality (Shapiro-Wilk W goodness of fit test, W = 0.97, P = 0.1). Over the three weeks of the experiment, average temperature decreased significantly (r =  −0.56, P<0.001), while average RH increased (r = 0.72, P<0.001).

**Figure 4:**
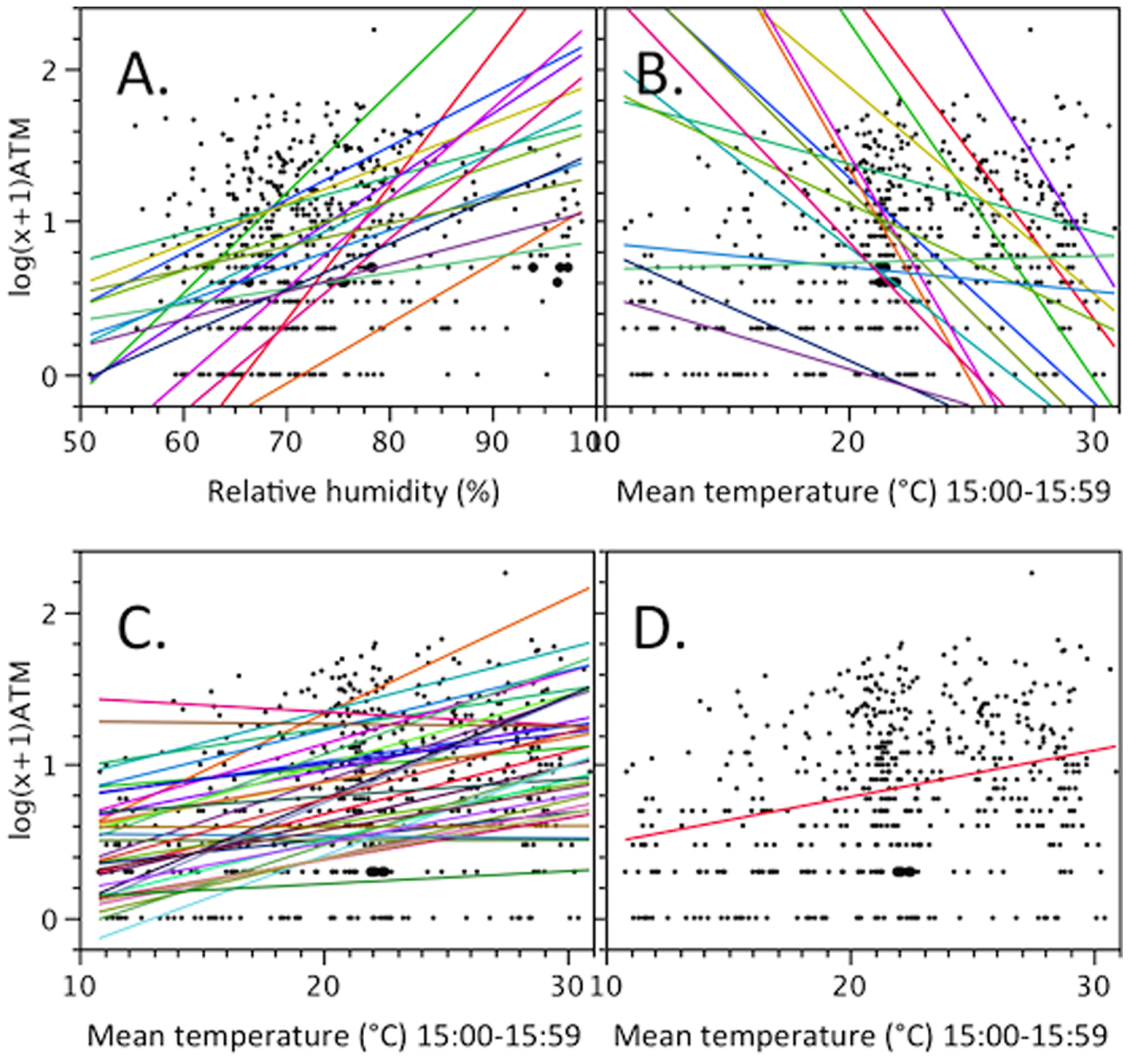
Relationship between trap catch (log+1 transformed) and temperature or relative humidity at local scale (A and B) and over time (C and D). Analyses of variance were nested within date in A and within trap in B. Graph D is a simplified version of C for better visualization. The color lines connect nested measurements (i.e. each color represent a trapping event in A and B and represent a specific trap in C).

Across BGS traps deployed for surveillance during the 2009 active season we found that cross-trap variability in temperature and RH was significantly higher in the afternoon than early in the morning (comparisons of matched standard deviations at each trap, t-ratio =  −11.3, P<0.0001). Therefore we restricted our analyses of the effects of temperature and RH on *Ae. albopictus* catches to the 15:00 data set. The results also indicate that in spite of our detailed guidelines for trap deployment, several traps were regularly deployed in significantly hotter and less humid environments than the average±95% confidence intervals (CI), indicating they were outliers. No outliers were recorded that were colder or more humid than the average±CI, indicating that most traps were placed in the shade. In addition, a repeated measures analysis did not reveal significant differences between experimental sites (Untreated, Education only, IPM) in average temperature or RH (MANOVA, F_2,16_ = 0.31, P = 0.13; F_2,16_ = 1.99, P = 0.17, respectively). Although the education and IPM strategies implemented during the season to reduce *Ae. albopictus* populations had a significant effect on local populations with reductions often over 75% [21], we found that irrespective of overall infestation intensity (i.e. in all 3 sites), during each trap event (time) *Ae. albopictus* catch was positively correlated with local humidity (Figure 4.A, overall P<0.0001). Also at each time, temperature was negatively and strongly correlated with local RH (average correlation±SD, r =  −0.84±0.16, all P<0.01) and consequently BGS catches were negatively correlated with temperature (Figure 4.B). In contrast, over the season (across trapping events) temperature and RH were not correlated (blocked by trap, r =  −0.02, P = 0.59), and the relationship between catches of *Ae. albopictus* and temperature at 15:00 was significantly positive (Figure 4.C and D, overall P<0.0001), while catch did not correlate with RH (not shown for brevity). To remain consistent with the among trap analysis, we restricted the seasonal analyses to the 16 weeks of the peak season. Nonetheless, the positive association between trap catch and temperature was even more dramatic if the analyses were expanded to the entire season, which included trapping in early summer and late fall (not shown for brevity).

## Discussion

Overall, our results indicate that BGS trap placement significantly affects the number of *Ae. albopictus* captured. The results were dramatic with catch rates in shade or partial shade over three times higher than those in areas exposed to the sun, which could strongly impact estimates of the size of the local adult mosquito populations and control decisions. The significant differences in trap catches between the treatments remained even as average air temperature and relative humidity (RH) changed over the three weeks of the experiment. This is an important result that should guide BGS trap placement for surveillance of *Ae. albopictus*. The BGS trap was designed specifically to attract day-biting *Stegomyia* mosquitoes that presumably rely to some extent on visual cues to find their hosts. In fact, this trap utilizes a highly contrasting black and white design that is thought to be essential to its success [26]. Our findings indicate that *Ae. albopictus* does not often venture into sunny areas, possibly because air temperature is higher or RH is lower in those exposed areas, as we observed. It is therefore possible that the effectiveness of this bright and contrasting trap results from its visibility in low light settings.

We were not surprised that the HOBO data loggers placed on top of the traps and therefore in some cases exposed to direct sunlight registered significant differences in temperature between sites in the same yard, but we were pleased by the degree to which Hygrochrons placed inside the traps were able to register consistent variation in temperature and humidity. The very strong correlation between light and temperature during each trapping event also indicates that at least at temperate latitudes such as in New Jersey we can use temperature measured by the Hygrochron as a proxy for light exposure. The results of the experimental approach were conclusive, and indicate that the specific location of a trap within a small yard can bias the results of BGS trap based surveillance programs for *Ae. albopictus*. Nevertheless it was the analyses of temperature and RH across a large array of BGS traps deployed within the City of Trenton for surveillance of *Ae. albopictus* that allowed us to explore in more detail the ways in which BGS catch can be directly impacted by trap location.

First, our results indicated that although there was a concerted effort to follow the guidelines to place all traps in similar conditions, there was significant variation across traps in local temperature and RH. Retrospective examination of the data indicated that outlier BGS traps were placed in yards where either no shade was available or conditions changed during the season (e.g. due to homeowner yard cleanup). It should be noted, that following agreed guidelines for the AW-ATM project, BGS traps were placed with prior homeowner permission within pre-established grids and in fixed locations over the entire season. It was therefore not always possible to place traps in the shade. As a result our analyses indicate that local temperature and RH affect trap catches: during each collection date, trap catches were negatively correlated with temperature and positively correlated with RH, as expected from the results of the field experiment on trap placement. This local effect contrasts with the positive correlation between trap catches and temperature over time (over the season), a common result that stems from the life-history of temperate mosquitoes, whose populations increase exponentially over the spring and summer and decline in the fall [27]. Interestingly, considering the strong negative correlation between temperature and RH observed locally, the expected negative correlation between *Ae. albopictus* catches and relative humidity does not occur over the season. Instead, the highest catches of *Ae. albopictus* across the whole season occurred at intermediate levels of RH (Figure 4.A). This indicates that *Ae. albopictus* is differentially collected in highest numbers when seasonal temperatures are high but in areas where local humidity is also high which are usually associated with lower temperatures. Importantly, the positive association between catch and RH during each trapping event held over the entire season (Figure 4.A) irrespective of mean air temperatures (Figure 4.A and B). This pattern may reflect either reluctance of adults to fly into or through dry areas [28] or local aggregation of adults in humid areas. If the first scenario predominates then traps placed in the sun will miss local populations of *Ae. albopictus*. If the second scenario dominates, however, catches from traps placed in full sun correctly reflect local *Ae. albopictus* abundance. However, the scale at which we demonstrated heterogeneity in catch rates (1–20 meters) is much smaller than the average dispersal capabilities of *Ae. albopictus* females [28], [29] or even males [30], which are in the order of 200–500 m. If adults aggregate locally, and they may, then the only way to quantitatively and accurately survey the adult populations of *Ae. albopictus* would be to deploy traps evenly across humid and dry (dark and bright) areas. Considering the cost and effort necessary to deploy large numbers of BGS traps [22], we propose that standardization of trap location with regards to temperature and humidity (or their proxy  =  light, at least in temperate climates) is a useful alternative. Our analyses also underscore the potential of even small expanses of exposed terrain, especially those that are relatively continuous such as a wide road or a highway, to become a barrier to the dispersal of this mosquito.

Mosquito surveillance is a prerequisite to an effective, efficient, and environmentally sound mosquito control program. Surveillance is used to define the nature and extent of the mosquito problem and to gauge daily mosquito control operations. It also provides the basis for evaluating the effectiveness of control operations and the transmission potential for mosquito-borne diseases. Thus the data collected needs to be reliable in order to justify and direct operations. Given the enormous effort undertaken to sample mosquito populations at numerous sites by mosquito control programs, ensuring that reliable data is being collected is critical. We have demonstrated that careful placement of BGS traps, in particular avoidance of locations exposed to direct sunlight, needs to be part of an effective surveillance program for *Ae. albopictus*, the Asian tiger mosquito.
